# Nanotechnologies: An Innovative Tool to Release Natural Extracts with Antimicrobial Properties

**DOI:** 10.3390/pharmaceutics13020230

**Published:** 2021-02-06

**Authors:** Umile Gianfranco Spizzirri, Francesca Aiello, Gabriele Carullo, Anastasia Facente, Donatella Restuccia

**Affiliations:** 1 Department of Pharmacy, Health and Nutritional Sciences Department of Excellence 2018–2022, University of Calabria, Edificio Polifunzionale, 87036 Rende, Italy; francesca.aiello@unical.it (F.A.); anastasiafacente_93@hotmail.it (A.F.); donatella.restuccia@unical.it (D.R.); 2 Department of Biotechnology, Chemistry and Pharmacy, Department of Excellence 2018–2022, University of Siena, Via Aldo Moro 2, 53100 Siena, Italy; gabriele.carullo@unisi.it

**Keywords:** nanotechnologies, plant extracts, agro-food-wastes, antimicrobial agents, polymeric nanocarriers

## Abstract

Site-Specific release of active molecules with antimicrobial activity spurred the interest in the development of innovative polymeric nanocarriers. In the preparation of polymeric devices, nanotechnologies usually overcome the inconvenience frequently related to other synthetic strategies. High performing nanocarriers were synthesized using a wide range of starting polymer structures, with tailored features and great chemical versatility. Over the last decade, many antimicrobial substances originating from plants, herbs, and agro-food waste by-products were deeply investigated, significantly catching the interest of the scientific community. In this review, the most innovative strategies to synthesize nanodevices able to release antimicrobial natural extracts were discussed. In this regard, the properties and structure of the starting polymers, either synthetic or natural, as well as the antimicrobial activity of the biomolecules were deeply investigated, outlining the right combination able to inhibit pathogens in specific biological compartments.

## 1. Introduction

Nanotechnology involves different strategies by using natural and synthetic materials in nanoscale dimensions to fabricate devices widely employed in the electronic and food industries, as well as in the pharmaceutical and biomedical fields [1]. Polymeric nanocarriers, due to their high surface area and small dimension (1–100 nm), are able to increase permeability and solubility of the enclosed molecules, making them available for several health applications, including diagnosis, disease treatments, and imaging [2,3,4]. In addition, effectively modifying the key features of nanocarriers, i.e., size, constituents, shape, and surface properties, it is possible to tune their mechanical, biological, and physicochemical characteristics [5]. In particular, nanotechnologies have gained outstanding consideration in the development of smart and effective pharmaceutical systems able to transport and deliver bioactive components in a specific site, avoiding, at the same time, deterioration due to enzymatic activity and pH values [6]. Among bioactive molecules, natural compounds have always represented the most widely employed substances for their unique therapeutic properties against several diseases [7]. In fact, natural bioactive extracts from plants, herbals, or agro-food by-products represent a rich source of compounds (polyphenols, anthocyanins, flavonoids, and many others) useful in the treatment of various diseases, thus suggesting their addition to pharmaceutical and cosmetic formulations, as well as to nutraceutical supplies [8]. In particular, many nanodevices (i.e., nanofibers and nanoparticles) have been developed to serve as antimicrobial agents to avoid pathogens’ proliferation. In literature, a large number of articles can be found, describing the transport of antimicrobial agents, mainly, but not only, to the skin compartment in the wound treatment, in order to prevent infections and/or to accelerate the healing process [9,10,11].

In this review, highly innovative nanotechnology-based delivery systems loaded with bioactive molecules recovered from natural matrices and showing antimicrobial activity were described. The referenced papers were selected through the articles published from the year 2010 and sorted based on the specific type of nanocarrier.

## 2. Natural Extracts with Antimicrobial Activity

The search for new therapeutically active compounds has spurred researchers over the years to investigate natural compounds [12,13,14,15,16,17]. In particular, food and plant wastes represent interesting sources of biologically active molecules [18,19,20,21,22,23,24] and have been proposed as indigenous remedies [25,26]. Specifically, secondary metabolites from plants represent valuable bioactive ingredients [27,28,29,30] with remarkable antibacterial properties [31,32], useful in the treatment of several diseases. Table 1 summarizes the main natural extracts proposed for their antimicrobial features.

The valuable therapeutic power of *Glycyrrhiza glabra* L. var cordara is well known: its extracts showed a panel of antibacterial features against various bacterial strains (128 < minimal inhibitory concentration (MIC < 512 µg/mL), the activity being mainly related to the pinocembrin, recovered in the extract as free and fatty acids-conjugated form [14]. Similarly, the nutritional properties of the male date palm flower, via defining its antibacterial actions, were also scouted [33]. The chromatographic analysis identified the presence of several phenolic compounds. Among them, quinic acid was recovered as the main component (84.52% *w/w*) and significantly influenced the nutraceutical and pharmacological properties of the extract.

Thin-layer chromatography (TLC) micro-fractionation of the organic extracts of *Ferula ferulioides* [34], a traditional medicinal plant, served as a guiding tool to isolate two compounds (dalpanitin and vicenin-3), with remarkable antimicrobial activity against drug-resistant *Staphylococcus aureus* [35].

Antimicrobial activity of essential oil (EO) of Cyprus *Citrus aurantium* L. flowers was analyzed, and the recorded minimum inhibitory concentrations (MIC) against Amoxycillin-resistant *Bacillus cereus* was 1.562 mg/mL [36]. Moreover, the extracts of *Psidium* sp., *Mangifera* sp., and *Mentha* sp. and its mixtures displayed antimicrobial effects and strongly reduced *Streptococcus mutans* [37].

The phytochemical study of the aerial part of *Pulicaria undulata* L. led to the isolation of nine compounds. The organic extracts (methanol, ethyl acetate, and dichloromethane) of the aerial parts were assayed by in vitro antimicrobial activity against a panel of sensitive microorganisms [38]. Similarly, the phenolic compounds in the solvent extracts of *Genista saharae* were analyzed. The chloroform extract, containing high amounts of quercetin and naringenin, revealed the antioxidant potential and antibacterial activity against the bacterial strains (MIC 0.02 mg/mL). These results seemed to indicate a high contribution of quercetin and naringenin in the antimicrobial and antioxidant activities recorded [39].

The prevalence of different types of chronic wounds due to the aging population and the increasing incidence of diseases is a worldwide clinical emergency. Various medicinal plants used in folk medicine and showing wound healing and antimicrobial properties have been widely assessed [40].

As known, some mushrooms and numerous other fungi exhibit innovative properties, including antimicrobial features against bacteria, fungi, and protozoans. In particular, in a study, 316 species of 150 genera from 64 fungal families were analyzed, showing antibacterial activity against different bacteria and fungi [41].

The main component of extracts from white guava (*Psidium guajava* L. cv. Pearl) was quercetin-glycosides. In particular, the micro-morphology of both *Escherichia coli* and *S. aureus* was changed with a flavonoids concentration of 5.00 mg/mL and 0.625 mg/mL, extracted from white guava leaves [42].

The traditional use of *Polyscias scutellaria* Fosberg to treat body odor suggested that this plant shows antibacterial properties. Most of the microorganisms hosted by human skin are harmless and even useful against pathogenic bacteria. Furthermore, *Acinetobacter* sp., formerly known as commensal bacteria, evolved into pathogenic bacteria and caused outbreaks in the intensive care unit. In this context, the antibacterial activity of *P. scutellaria* Fosberg extracts against *Acinetobacter* sp. isolated from healthy human armpit was investigated [43].

The ethyl acetate fraction from the leaves of *Schismus fasciculatus* contained kaempferol, quercetin, and agathist flavone, which showed moderate antibacterial activity against different tested strains (IC_50_ 0.9 mg/mL) [44]. In addition, both cardamom (*Elettaria cardamomum*) fruit and seed extracts exerted remarkable antibacterial effect against *Aggregatibacter actinomycetemcomitans*, *Fusobacterium nucleatum*, *Porphyromonas gingivalis*, and *Prevotella intermedia* [45].

The antimicrobial properties of oregano (*Origanum vulgare*), sage (*Salvia officinalis*), and thyme (*Thymus vulgaris*) essential oils (EO) were assayed against *Klebsiella oxytoca* (MIC of 0.9 mg/mL for oregano EO and 8.1 mg/mL for thyme EO) [46].

The water and ethanolic extracts of *Myristica fragrans* (Myristicaceae) wood displayed interesting antimicrobial, anti-inflammatory, and antioxidant activities [47].

The phenolic composition, antimicrobial activities, and antioxidant activity of *Euphorbia tirucalli* L. extracts were evaluated by agar dilution methods, and MIC values were recorded. In all samples, ferulic acid resulted as the main phenolic compound identified and quantified through LC-UV. The extracts demonstrated inhibitory potential against *Staphylococcus epidermidis* and *S. aureus* [48].

The methanol leaf extract of *Tradescantia zebrina* showed the highest antioxidant content and activity, exhibiting antibacterial activity against six species of Gram-positive and two species of Gram-negative bacteria in a range of 5–10 mg/mL [49].

The aqueous extracts of *Adiantum caudatum* leaves, obtained by Soxhlet extraction, resulted as more powerful than the hexanoic one against *Pseudomonas aeruginosa* [50]. The methanol extracts from the flowers of *Agastache rugosa* (Korean mint) showed high antibacterial activities [51]. Finally, the carotenoid fucoxanthin was observed to have a significantly stronger impact on Gram-positive than Gram-negative bacteria [52,53].

## 3. Agro-Food Wastes as Antimicrobials

The processing of agro-products generates huge amounts of waste materials every year in the form of peels, seeds, and oilseed meals, thus representing serious environmental concerns. Besides, the cost of drying, storage, or transportation poses a severe financial limitation to wastes utilization. To support the transformation and exploitation of these by-products, there is a growing interest in recycling waste biomass of agro-products in particular, considering their therapeutic properties (Table 2).

For example, the major wastes for industrial apple juices are the seeds. After a Soxhlet extraction, oil was recovered, and this apple seed oil was completely active against bacteria, showing MIC values ranged from 0.3–0.6 mg/mL [54].

About a quarter of the total tomato production undergoes processing, leading to derivatives like sauces, canned tomatoes, ketchup, or juices, largely consumed worldwide. At the same time, the tomato industry generates huge quantities of wastes, up to 5–0% of the total production. These by-products are used as livestock feed or discarded in landfills, creating many environmental problems. However, considering that valuable phytochemicals, such as carotenoids, polyphenols, tocopherols, some terpenes, and sterols, resist industrial treatment, tomato by-products represent also a precious resource. An interesting experimental work reported that the most active peel tomato extract against *S. aureus* and *Bacillus subtilis* (MIC: 2.5 mg tomato peels/mL) belonged to the Ţărăneşti roz variety, owing to its high carotenoid amount [55].

Fennel and carrot, two species belonging to the Apiaceae family, are, like many others (e.g., tomatoes, potatoes, and onions), the most commonly consumed vegetables worldwide. They are aromatic and have been used as spices and condiments. Their EO, related to the fruits, is well characterized, whereas the chemical composition of the leaves, a by-product, is poor. Wiem Chiboub and co-workers performed a hydrodistillation of fresh leaves of carrot and Daucus carota subsp. sativus orange roots and yellow roots and *F. vulgare* subsp. vulgare var. azoricum and *F. vulgare* subsp. vulgare var. latina. The recorded results showed that the Daucus carota subsp. sativus yellow roots oil was significantly more effective against Gram-negative than Gram-positive bacteria, and the MIC values were in the range 6.25–50 mg/mL [56].

In order to reuse agro-wastes, the betel leaf stalk extract was found to be a potent antimicrobial agent, showing activity against Gram-positive and Gram-negative bacteria. The MIC values were in the range 25–250 μg/mL, measured against ciprofloxacin as a standard [57].

Among agro-food wastes, those derived from the olive oil production represent the most representative, especially in the Mediterranean area. Their composition was found to be rich in hydroxytyrosol and secoiridoids derivatives, important for their healthy properties. Inass et al. investigated the in vitro antimicrobial potential of olive mill wastewater and olive cake extracts. Oleuropein and verbascoside, already pointed out in various studies for their important antimicrobial potential, were also detected in these extracts. Furthermore, the elenolic acid, the main fragment of the oleuropein degradation, was mostly found in the olive cake extract. It can be considered as an important antimicrobial and antiviral agent, justifying the reuse of this kind of wastes [58].

Besides, the fruits belonging to the Citrus sinensis family produce large amounts of wastes, mostly seeds and peels, endowing suitable biological value. Seed oil demonstrated better activities than peel oil, with remarkable inhibitions obtained against *S. aureus* and *Candida albicans* at a concentration as low as 2.5 mg/mL [59]. Furthermore, the orange peel of 12 cultivars of Citrus sinensis from central-eastern was extracted through steam distillation and using hexane. In all the cultivars, the main component was d-limonene (73.9–97%). The antimicrobial activity was investigated against *S. aureus*, *Listeria monocytogenes*, and *P. aeruginosa*. ‘Sanguinello’ and ‘Solarino Moro’ essential oils were significantly active against *L. monocytogenes*, while ‘Valencia’ hexanoic extract against all the tested microorganisms [60].

In this context, the winemaking process is also involved in smart wastes management. Grape seeds are the by-products of the fruit juice and wine industries. Nowadays, more attention is devoted to the valorization of these kinds of wastes due to the valuable phytochemicals content. A study performed on different varieties of grape seeds, extracted with 70% ethanol, showed that all the tested varieties possessed a considerable antibacterial activity. Particularly, the variety Shiraz showed a large zone of inhibition (17 mm) [61]. Different fractions of wine residue (pomace, including seed and skin, seeds, or skin) from two red varieties of *Vitis vinifera* grapes (Pinot noir and Pinot Meunier) grown in New Zealand were extracted using different water-organic solvent mixtures. It was found that all the extracts exhibited antibacterial and antifungal effects, with MIC values ranging between 0.195 and 100 mg/mL [62].

Similarly, lavender and melissa wastes were proved to be rich in polyphenols (especially rosmarinic acid) and exhibited high antimicrobial activity [63]. The processing of jackfruit (*Artocarpus heterophyllus* Lam) yields large amounts of bio-wastes. The ethyl acetate extracts obtained from the peel, fiber, and core of the jackfruit showed antibacterial activity against *Xanthomonas* axonopodis pv. manihotis [64].

Apple and Sabine mango kernel extracts exhibited significantly high inhibition zones of 1.93 and 1.73 compared to Kent and Ngowe with 1.13 and 1.10, respectively, against *E. coli*. For *C. albicans,* the inhibition of Kent mango kernel extract, 1.63, was significantly lower than that of Ngowe, Apple, and Sabine with 2.23, 2.13, and 1.83, respectively [65].

The *Vaccinium meridionale* Swartz pomace is a source of bioactive compounds with remarkable antibacterial activity. Quercetin derivatives represented 100% of the total flavonols in the extracts, and *S. aureus* was the most sensitive strain [66].

The walnut green husk is an agro-forest waste obtained during walnut (*Juglans regia L.*) processing; its aqueous extracts were found to be able to inhibit the growth of Gram-positive bacteria [67]. Another study reported the antioxidant and antimicrobial activities of extracts of pecan nutshell. The MIC and minimum bactericidal concentration (MBC) values against *L. monocytogenes*, *Vibrio parahaemolyticus*, *S. aureus*, and *B. cereus* were significantly lower (*p* < 0.05) for the extract obtained through infusion, followed by atomization in a spray dryer when compared to the other extracts [68]. Water, methanol, ethanol, and 50% (*v/v*) aqueous solutions of methanol and ethanol extracts of disposed garlic husk displayed antimicrobial activity against Gram-positive bacteria when applied at different concentrations (1–10 mg/mL). These interesting biological properties could be attributed to specific phenolic compounds, such as caffeic, p-coumaric, ferulic, and di-ferulic acids [69].

Extracts prepared from mangosteen bark or fruit pericarp exhibited strong pH-dependent bacteriostatic and bactericidal effects against *L. monocytogenes* and *S. aureus* [70]. Ethyl acetate extract of Newhall orange peel showed the best antimicrobial effect due to the presence of sinensetin, 4′,5,6,7-tetramethoxyflavone, nobiletin, 3,3′,4′,5,6,7-hexamethoxyflavone, and narirutin [71]. Punica granatum peels (Bhagwa) furnished extracts, endowing interesting antibacterial properties. LC analysis of the extract recorded the punicalagin (163.52 mg/g of waste) as a major ellagitannin compound. Peel extract exerted high antibacterial activity against both Gram-positive and Gram-negative bacteria [72].

Extracts from brewer’s spent grain, the major by-product of the brewing industry, proved to be a rich source of bioactive compounds with antimicrobial activity (especially against *C. albicans*) [73].

The phytochemical screening of the aqueous extract of the Agave sisalana Perrine juice (waste) revealed the presence of saponins, glycosides, phlobatannins, terpenoids, tannins, flavonoids, and cardiac glycosides and had the potential to be used against pathogenic organisms [74].

The antimicrobial activity of three abundantly available fruits peel waste (orange, yellow lemon, and banana) was evaluated on a wide range of microorganisms. Methanol, ethyl acetate, ethanol, and distilled water were used for extraction, and the results showed that, among the used solvents, the extracts exhibiting better performances were in decreasing order: Distilled water > Methanol > Ethanol > Ethyl acetate, reflecting the suitability of solvent for fruit peel extraction. Additionally, the effectiveness of fruit peel extracts was evaluated, showing Yellow lemon > Orange > Banana peel. It was observed that Gram-negative bacteria were more sensitive to the extracts, and, among them, *Klebsiella pneumoniae* showed the highest sensitivity against the extract of yellow lemon peel with the highest zone of inhibition [75]. Beet stalk, peanut peel, Pinot Noir grape marc, Petit Verdot grape seed and marc, red grapes fermentation lees, and guava bagasse wastes showed antimicrobial activity against *S. aureus* and *L. monocytogenes*. Analyses by GC-MS identified relevant concentrations of compounds exhibiting antimicrobial activity, such as caffeic, gallic, ferulic, and r-coumaric acids, and flavonoids quercetin, myricetin, and epicatechin. This study confirmed that agro-industrial wastes from wine and food industries could be used in the research about new antimicrobial compounds to be used as natural preservatives in the food and beverage industry with promising applications also in the pharmaceutical and biomedical fields [76].

With a growing world production, mango represents one of the most important tropical fruits produced worldwide. India is one of the most important producers where any mango-based products are also commonly consumed. Mango is mostly used in food processing industries, such as juice, jam, jelly, and pickle industries. This processed food leads to an enormous generation of mango peel as a waste product. It needs a huge capital to decompose these peels and to make sure that it does not pollute the environment. The EO of both mango indica cultivars pulp and peel showed a wide range of antibacterial and antifungal activities [77].

The peel of Camu-camu (*Myrciaria dubia* (Kunth) McVaugh) displayed the richest phenolic profile as well as the most significant antibacterial activity (MICs recorded were in the range 0.625–10 mg/mL) [78].

Interesting results showed that the fraction with the highest content of phenolic and secoiridoid compounds from crude olive mill wastewater had a relevant antibacterial activity against a large panel of strains with a strain-dependent character [79].

The natural food colors market is trying to fit the consumer needs by increasingly replacing synthetic additives with natural ones. Besides being a natural product, the biological relevance of the carotenoids is related to their potential antioxidant and antimicrobial features, boosting their wide application in the food and pharmaceutical industry. The production of carotenoids by microorganisms using agricultural waste has been reported, employing coffee pulp and husk using a non-conventional yeast [80]. Despite the high antimicrobial activity exhibited by these extracts, their bioavailability is poor when used as such. In this context, the inclusion of nanocarriers seems to be an innovative tool to overcome this limit.

## 4. Nanofibers as Carriers of Antimicrobial Natural Products

**Figure 1:**
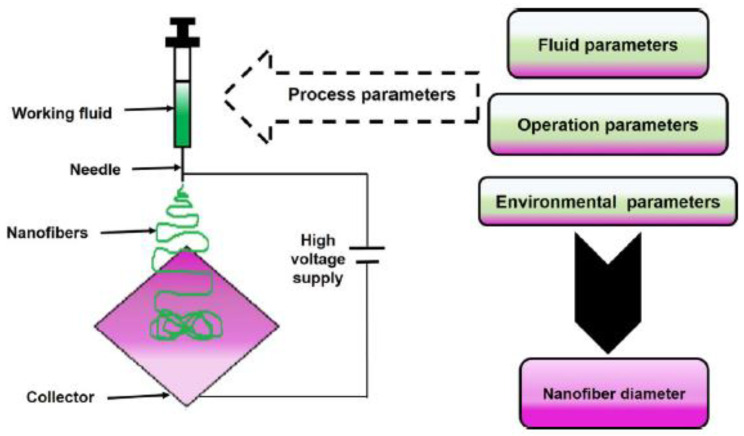
Electrospinning process and the different experimental parameters affecting the diameters of the produced nanofibers. Reproduced with permission from [10], Elsevier, 2020.

During the last years, an increasing interest in the biomedical use of polymeric nanofibers was recorded due to their high porosity, outstanding mechanical strength, and simplicity of fabrication [10]. In particular, nanofibers have been proposed as systems for the delivery of bioactive molecules [81] or in regenerative medicine [82] and also as wound dressings devices [83]. Usually, polymeric nanofibers can be fabricated by template synthesis [84], self-assembly [85], phase separation [86], and electrospinning [87]. Among these, the electrospinning technique is the most used strategy for applications in tissue engineering and drug delivery due to its high-throughput, easy handling, and reproducibility (Figure 1).

**Figure 2:**
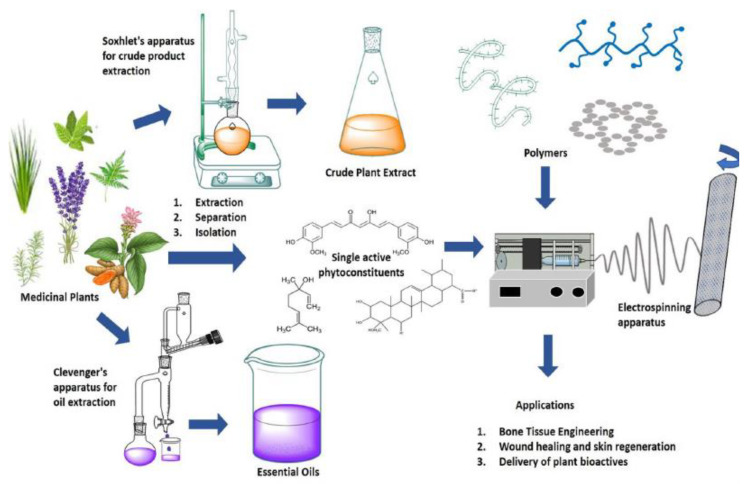
Schematic representation for the use of extracts isolated from medicinal plants and their nanofibers fabrication useful for pharmaceutical and biomedical applications. Reproduced with permission from [11]; Elsevier, 2020.

Additionally, electrospun meshes significantly increase adhesion and drug loading due to the structural similarity to the extracellular environment of the living tissues [88]. Literature data clearly indicate that electrospun nanofibers have the remarkable potential to amplify effectively the biological properties of medicinal plant extracts, essential oils, or pure single components with antimicrobial features. For this reason, they were largely employed as bioactive molecules to fabricate delivery systems, tissue engineering scaffolds, and regenerative medicine devices (Figure 2) [11].

Specifically, in order to obtain polymeric nanofibers able to protect bioactive molecules and to ensure their controlled and site-specific delivery, both synthetic (polyesters, polyvinyl pyrrolidone (PVP), polyvinyl alcohol (PVA), or polyacrylates) and/or natural (polysaccharides or proteins) macromolecular structures have been proposed [89,90]. Synthetic constituents are usually cheaper and stronger, more easily electrospinnable, and show a precise structure [91]. Among the synthetic polymers used in the preparation of the nanofibers, PVA, PVP, and polyesters, such as polylactic, polylactic-*co*-glycolic acid, polyurethane, and polycaprolactone, were widely used with significant results (Table 3). On the contrary, natural polymers can be obtained by environment-friendly sources, offer good biocompatibility, thus reducing the adverse effects that could be observed when introduced to the human body [65] (Table 4). However, synthetic polymers are often required to strengthen the weak mechanical resistance of natural polymers [66].

### 4.1. Synthetic Polymer-Based Nanofibers

#### 4.1.1. Polyacrylates

Among polyacrylates, polyacrylonitrile (PAN) has been well documented to be a very important material for easy manufacturing of synthetic fibers with unique thermal and mechanical stability, as well as excellent resistance to the solvents [144,145]. In the biomedical field, PAN nanofibers loaded with moringa leaf extracts, rich in phytochemicals, such as zeatin, quercetin, amino acids, and phenolic compounds, have been proposed for wound healing applications [92,146]. The antibacterial properties of the loaded nanofibers were evaluated against *E. coli* and *S. aureus*. Results displayed that the activity of the device strictly depended on the concentration of the extract in the PNA nanofibers, confirming its promising potential as an effective wound dressing. Alternatively, electrospinning PNA nanofibers loaded with *Syzygium aromaticum* oil, containing unexplored natural bioactive molecules (eugenol and caryophyllene), were found to be highly effective against both Gram-positive and Gram-negative bacteria in in vitro delivery studies [93,147]. The release profile of *Syzygium aromaticum* was characterized by an initial burst, followed by controlled diffusion kinetics. Similarly, PNA fibers were loaded with EO of lavender (*Lavendula angustifolia*) [94]. This natural product is largely exploited in aromatherapy as a holistic relaxant and has been exploited for its excellent antimicrobial features. Lavender oil, highly encapsulated in PAN fibers with a loading content equal to 13.6%, displayed a release profile with an initial burst effect, reaching a 35% of active release after 24 h in a buffer medium (pH 7.4). The loaded polymeric device showed also in vitro antibacterial activity after an incubation time of 24 h (MIC equal to 100 mg·mL^−1^).

#### 4.1.2. Polyesters

Poly (lactic acid) (PLA) is an outstanding polymeric material largely employed in the pharmaceutical and biomedical sectors due to its excellent biodegradability and biocompatibility [148]. PLA represents the most extensively used synthetic polymer in the fabrication of nanofibers [149]. Electrospinning of PLA fibers containing EO derived from *Leptospermum scoparium* and *Melaleuca alternifolia* at different concentrations has been proposed for the treatment of microbial infections induced by *S. epidermidis* [95], a bacterium abundant on human skin and often responsible for infections and formation of biofilms on the medical devices [150]. The antibacterial activity of *Leptospermum scoparium* EO was mainly ascribed to triketone species, such as flavesone and leptospermone [151], while terpinen-4-ol and α-terpinene determined the antimicrobial activity of *Melaleuca alternifolia* EO. Although *Melaleuca alternifolia* oil showed remarkable activity against *S. epidermidis*, its efficacy was largely limited due to the low amount of active molecules available after the electrospinning process. On the contrary, the antimicrobial activity of the PLA-*Leptospermum scoparium* oil system was preserved during the electrospinning process.

In order to improve its water solubility, PLA was successfully modified by hyperbranched polyglycerol, a highly hydrophilic polymer with excellent biocompatibility and water solubility [152]. Nanofibrous dressing with PLA and the hyperbranched polyglycerol blend was fabricated using electrospinning technique and loaded with curcumin, a polyphenolic biologically active ingredient of turmeric isolated from the dry rhizomes of *Curcumin Longa* L. [153]. Curcumin release profiles, recorded at 37°C in PBS and displayed as the increased hydrophilicity of the device, allowed to achieve a complete delivery in 72 h, suggesting its use as potential wound patch dressing for chronic and acute wound diseases.

Alternatively, fibers based on poly(d,l-lactide-*co*-glycolide) (PLGA) have been successfully proposed as delivery systems due to their high biodegradability. In particular, the electrospinning technique was employed to fabricate ultrafine PLGA fiber mats containing a methanolic extract of *Grewia mollis* (7.5% *w/w*). The antimicrobial effect of the device was tested against pathogenic bacteria, such as *E. coli* and *S. aureus*, suggesting its use in the treatment of dermal bacterial infections or as a wound dressing agent [96]. Garcia-Orue and coworkers developed a PLGA-based nanofibrous membrane in which the antibacterial properties of the natural extract and the ability of the epidermal growth factor to enhance fibroblast proliferation allowed the fabrication of an efficient device against *S. aureus* and *S. epidermidis*, reducing, at the same time, the wound healing time [97].

Polycaprolactone (PCL) represents a biodegradable aliphatic polyester, largely employed to produce nanofibers by electrospinning techniques. The incorporation of suitable natural extracts allowed the fabrication of devices with remarkable antimicrobial properties. Specifically, crude extract of *biophytum sensitivum* was loaded, and experimental release displayed a controlled delivery of the bioactive molecules (59% in 72 h) [98]. The antimicrobial performances of the device were evaluated against some common wound bacteria, such as *S. aureus* (27 mm inhibition) and *E. Coli* (47 mm inhibition). Similarly, electrospun PCL-based nanofiber mats containing natural leaves extracts of *Gymnema sylvestre* [99] or *Clerodendrum phlomidis* [100] were proposed as a wound dressing with remarkable antimicrobial characteristics. The cumulative delivery profiles depicted an intense burst effect, mainly due to bioactive desorption release mechanisms, while a constant delivery of the therapeutic was reached after 24 h.

*Althea officinalis* extract is also well known as a traditional herbal drug with noteworthy wound healing capacity and antimicrobial properties. In particular, *Althea officinalis* extract was loaded in nanofibers based on PCL and gelatin [101]. The blending of synthetic and natural macromolecules was proposed as an efficient strategy to optimize the mechanical properties of the scaffolds. In addition, the superior viscoelastic properties of PCL with respect to other polyesters made it more compatible with natural macromolecules, allowing the construction of multilayer structures. Cumulative release experiments highlighted a release of bioactive molecules proximately to 100% after 24 h. Similarly, the PCL layer was deposited onto the gelatin films by electrospinning to obtain complex structures able to vehicle black pepper oleoresin bioactive components. Strong antimicrobial properties of the multilayer were recorded over time (10 days) against *S. aureus* [102].

Blended polymer-based electrospun nanofibers have gained great attention because of the mechanical properties of the matrices and easy monitoring of the release profiles, depending on the ratio of the components in the polymer chains. By coupling the strength of PCL with polyvinyl pyrrolidone (PVP), PCL/PVP nanofibers containing crude bark extract of *Tecomella undulata* were fabricated, and their antimicrobial behavior was evaluated against *S. aureus*, *E.coli,* and *P. aeruginosa* [103]. Recently, in order to improve antimicrobial properties of PCL-nanofibers loaded with *Nephelium lappaceum* extract, they were decorated with silver nanoparticles [154], providing a multi-component device with synergistic antibacterial properties [104]. The antimicrobial activity was evaluated against *E. coli*, *S. aureus*, and *P. aeruginosa,* and the results suggested a synergistic effect between silver nanoparticles and extract-loaded nanofibers.

Polyurethane (PU) is a biocompatible hydrophobic polymer, largely used for biomedical and pharmaceutical scopes, owing to its excellent oxygen permeability and good barrier and mechanical properties [155]. Nanofibers based on PU were successfully prepared to load emu oil, a natural mixture derived from the emu (*Dromaius novaehollandiae*), which originated in Australia and positively tested against both Gram-positive (*B. subtilis*) and Gram-negative (*E. coli*) pathogens [105]. Similarly, PU nanofibers were proposed as polymeric carriers of propolis [84], a resinous substance produced by bees from plant exudates, containing different bioactive compounds [156]. In addition, the adhesive properties of propolis afford the point-bonding to the PU nanofibers, improving their mechanical issues. The antimicrobial properties of propolis/PU nanofibers were evaluated against *E. coli*, and the results showed that the bacterial inhibition zone was progressively increased with increasing the propolis amount in the nanocomposite. More recently, herbal extracts of *Agrimonia eupatoria*, *Satureja hortensis*, and *Hypericum perforatum* were loaded on several PU composite nanofibers, and their antimicrobial properties were evaluated against *S. aureus* and *P. aeruginosa* [110]. Finally, antibacterial and elastic nanofibers from thermoplastic PU were produced by coating process with *Syzgium aromaticum* extract, gained by Soxhlet extraction of clove oil [107]. All coated nanofibers (2–10 mg cm^−2^) showed antibacterial activity against *S. aureus* and *E. coli*.

Nanomaterials to be employed as antimicrobial clothing materials or bedding materials for allergic patients often required a lamination process. In this regard, the nanofibers of PU/*Juniperus chinensis* extracts were prepared by laminating electrospraying PU adhesive resin on polyethylene terephthalate-based fabric and an electrospun PU nanofiber web [108]. The antibacterial experiments performed at 110 and 130°C against *S. aureus* and *K. pneumoniae* confirmed that the combination between laminating and electrospinning represents an interesting way to project useful composites to be employed in the biomedical field. Finally, in order to improve the absorption ability of wound exudates, hydrophilic blend materials based on a different weight ratio between PU and carboxymethyl cellulose (CMC) and containing *Malva sylvestris* extract were prepared. The new materials proved to efficiently deliver the bioactive compounds to diabetic wounds [109]. The release profile of the active compounds resulted in a complete delivery in 85 h, and strong antibacterial activity against *E. coli* and *S. aureus* was recorded.

#### 4.1.3. Poly(Vinyl Alcohol)

Synthetic biodegradable polymers, such as PLA, PLGA, and poly(glycolic acid), are poorly soluble in water. It follows that the production of nanofiber mats often requires the use of organic solvents, incompatible with the preparation of carriers for pharmaceutical and biomedical uses. On the contrary, biocompatible, biodegradable, and no-toxic poly(vinyl alcohol) (PVA) can be easily employed to prepare nanofibers in aqueous environments [157]. Different natural extracts, as sources of antimicrobial molecules, were enclosed in PVA nanofibers. In particular, *Lawsonia inermis* leaves’ hydroalcoholic (ethanol/water 90/10 *v/v*) extracts were loaded to achieve antibacterial nanofibers (2.8% *w/w*) with bacteriostatic action towards *E. coli*. and bactericidal efficiency against *S. aureus* [110]. Similarly, the methanolic extracts of *Tridax procumbens* leaves [111] and the aqueous extracts of *Coptis chinensis* [112] were employed to prepare nanofibrous mats with antimicrobial activity. PVA/*Tridax procumbens* nanofibers showed an outstanding zone of inhibitions and improved resistivity power against *S. aureus* and *E. coli*, whereas PVA/*Coptis chinensis* nanofibers, evaluated against different Gram-positive bacteria, displayed the highest antibacterial activity against *S. epidermidis*. The ethyl acetate *Rhodomyrtus tomentosa* extract, rich in myricetin and rhodomyrtosone, was involved in the fabrication of electrospun nanofibers; the antimicrobial experiments against the common human pathogens (*E. coli*, *P. aeruginosa*, *B. subtilis,* and *E. faecalis*) displayed a clear inhibition zone (7–12 mm) by loading the extract in the concentration range of 1.5–2.5% (*w/w*) [113]. Finally, nanofibers containing *Coptidis rhizoma* extracts (10, 20, and 30% *w/w*) were fabricated using PVA as a carrier [114]. The release experiments performed at physiological pH of 5.5, set by acetate buffer solution, displayed an initial fast release, followed by a gradual release for 48 h. In addition, high antimicrobial activity was recorded against *S. aureus* (max inhibition zone 17.0 mm) and *S. epidermidis* (max inhibition zone 6.2 mm).

In addition, PVA was employed in combination with a natural polymer to achieve bio-nanocomposites fibrous mats. Specifically, a PVA-based device enclosing nanocellulose from pineapple was loaded with *Stryphodedron barbatimao* extract (water-alcohol solution 96:4 *v/v*) [115], a well-known mixture employed in medicine as an antiseptic, anti-inflammatory, and anti-bacterial remedy [158]. More recently, PVA/guar gum composite nanofibers were proposed as a carrier of alcoholic extract of *Acalypha indica*, a traditionally acclaimed plant for wound healing [116]. The release profiles displayed a constant and slow delivery throughout the experimental time, while a complete release was observed after 38 h. The antimicrobial experiments against *E. coli, B. subtilis, S. aureus, P. fluorescens* highlighted bacteriostatic action against all microbial strains. Finally, several EOs (cinnamon, clove, and lavender at a concentration of 0.5, 1, and 1.5% *w/w*) were involved in the synthesis of PVA/sodium alginate (SA) polymeric nanofibers, allowing the preparation of devices able to efficiently inhibit *S. aureus* [117]. Cinnamon oil (1.5% *w/w*)/PVA/SA nanofibers exhibited the best results (inhibition zone of 2.7 cm).

#### 4.1.4. Polyvinylpyrrolidone

Polyvinylpyrrolidone (PVP) exhibits unique properties because it is biocompatible, water-soluble, and non-toxic, allowing its application in the biomedical area [159].

*Sophora flavescens* extract was incorporated into PVP nanofibers, and the device was proposed as antimicrobial air filtration [118]. Filtration and antimicrobial performances of the PVP-based nanofiber filter were evaluated, employing *S. epidermidis* bioaerosols as test airborne particles; the results displayed excellent antimicrobial activity and highly effective air filters (99.99% filtration efficiency).

Nutraceutical properties of the cinnamon EO were exploited by enclosing the oil in the PVP-based nanofibers by oil-in-water emulsion electrospinning, and its antimicrobial properties were recorded against *S. aureus, E. coli, P. aeruginosa, C. albicans* [119]. The outstanding antimicrobial activity of the device was proved to be related to the size of the fibers and mainly ascribed to the high eugenol concentration in the cinnamon oil. Experimental tests were highlighted as the antimicrobial activity was recorded with cinnamon EO concentration into PVP in the range 2–4% (*w/w*).

### 4.2. Natural Polymer-Based Nanofibers

#### 4.2.1. Polysaccharides

Materials based on cellulose acetate (CA) have been largely employed in the biopharmaceutical processing industry as wound dressings, tissue engineering scaffolds, and drug delivery systems [160]. Electrospun CA nanofibers encapsulating lemongrass, cinnamon, and peppermint EOs were employed to fabricate scaffold devices able to inhibit bacteria growth. Results demonstrated the complete inhibition of *E. coli*, while *C. albicans* yeast showed a remarkable resistance, mainly due to its diameter more than four times larger than *E. coli* [120]. More recently, CA-based nanofibers enclosing rosemary and oregano EOs were evaluated against *S. aureus*, *E. coli,* and *C. albicans* [121]. Nanofibers loaded with the oregano oil (containing a high concentration of carvacrol and thymol) displayed the best anti-biofilm and antimicrobial performances. Electrospun nanofibrous mats based on carboxymethyl cellulose (CMC) and SA were proposed as functional wound dressing materials able to load olive leaf extracts [122]. The antibacterial properties of oleuropein and hydroxytyrosol contained in this aqueous extract allowed to fabricate a device able to inhibit pathogens mostly responsible for infections by skin wounds. The analysis of the release profile from the nanofibers depicted a substantial burst effect (50%) and a complete delivery within 24 h.

Nanofibers have also been proposed as reinforcing agents to improve the mechanical and physical performances of other polymeric devices, such as films, hydrogels, and sponges [161]. Nanocomposites based on CMC and cellulose nanofibers (CNF) were loaded with *Salvadora persica* hydro-alcoholic extract to obtain a device with good antimicrobial properties against both *S. aureus* and *E. coli*. [123]. The incorporation of CNF (5% *w/w*) into the CMC matrix caused a significant improvement of the mechanical properties in comparison with CMC. Similarly, composite biosponges of SA reinforced with CNF were prepared [124], determining a noteworthy enhancement in the mechanical performances and the thermal stability for SA-based composite biosponges. Moreover, the sponges loading with *Oryza sativa* and *Tinospora cordifolia* extracts imparted additional antibacterial functionality to the composite against *E. coli* and *P. aeruginosa*.

The encapsulation into nanofibers of the EOs gained from natural plants often requires a strong electric field and high-voltage. This could lead to adverse reactions, frequently involving the species able to impart pharmacological and nutraceutical properties to the extracts. To overcome this inconvenience, the employment of the β-cyclodextrin represents an endearing strategy [162]. *Litsea cubeba* EO was first encapsulated in the cavity of the β-cyclodextrin, and the inclusion complex was then loaded by electrospinning into nanofibers based on the dandelion polysaccharide [125], a natural polymer used for the preparation of active nanofibers [163]. The release profiles of the EO in PBS solution (pH 7.2) showed a good sustained release (about 70% after 100 h), ensuring also a long-lasting antibacterial effect against *S. aureus*.

Biomedical devices based on chitosan (CT) were exploited for developing nanofibrous wound dressing materials [164]. Additionally, CT is also known for its strong antibacterial activity against different fungi, bacteria, and viruses [165,166,167]. However, CT viscosity, as well as the high charge of the polysaccharide chains, made difficult the electrospinning process, and the application of highly acidic and toxic solvents was required [168]. These drawbacks can be overcome by co-spinning CT with other spun polymers, such as poly(ethylene oxide) (PEO), PCL, and PVA [169].

CT-ethylenediaminetetraacetic acid and PVA were selected for the preparation of nanofibrous mats to be employed as carriers of extracts gained by maceration (acetone/water 70/30 *v/v*) of *Garcinia mangostana* fruit hull [126], known to exert important antimicrobial properties due to the presence of the α-mangostin (13.20% *w/w*) [170]. α-Mangostin release experiments from the nanofiber mats loaded with the extracts revealed that active molecules were rapidly released, reaching 80% within 1 h. Additionally, the fiber mats loaded with the natural extracts exhibited antibacterial activity against *S. aureus* and *E. coli*. More recently, CT and PVA were successfully used to fabricate electrospun nanofibers containing *Bidens pilosa* extract. Composite nanofibers were effective against *E. coli* (growth inhibition 91% and MIC 10 mg/mL) and *S. aureus* (growth inhibition 86% and MIC 10 mg/mL) bacteria due to the combined effect of CT and natural extract [127]. Polymeric devices with multi-antibacterial activity were prepared by electrospinning of CT and honey, a carbohydrate-rich syrup, showing a relevant wound healing activity and remarkable antibacterial performances [171]. The synthesis of honey-based nanofibers was frequently hard due to the viscosity of the honey that permits its use in the electrospinning process only at small concentrations (<10%) [172]. Electrospinning of CT and honey at high concentration, with PVA and using eco-friendly solvents, was performed, and the antimicrobial activity of the fiber mats was evaluated against *E. coli* and *S. aureus* [128,129]. In addition, two aqueous extracts (*Cleome droserifolia* and *Allium sativum*) were loaded within honey, PVA, and CT nanofibers; in vitro antimicrobial experiments revealed a complete inhibition of *E. coli* and *S. aureus* [130]. However, only the fibers simultaneously loaded with both the extracts exhibited some antimicrobial activity against methicillin-resistant *S. aureus*. In addition, preliminary in vivo study (wound closure rates in mice and histological examination of the wounds) revealed the beneficial effects of the extract-loaded device on the wound healing process in comparison with the untreated control.

Alternatively, CT-based nanofibers can be fabricated employing PEO, which allows the use of solutions at pH 4, thus not requiring an extremely acid environment. CT/PEO solutions were successfully electrospun, by oil-in-water emulsion technique, into fibrous mats and loaded with cinnamaldehyde (0.5 and 5.0% *w/w*), a volatile EO derived from cinnamon bark [131]. The delivery/antimicrobial experiments of cinnamaldehyde EO from the CT/PEO nanofibers displayed a strong inactivation of *P. aeruginosa* (81% after 180 min). Finally, a green tea extract was proposed as an antibacterial enhancer to be enclosed in the electrospinning process involving CT/PEO chains [132]. In vitro tests revealed that this device had an antibacterial effect against *E. coli* and *S. aureus*.

#### 4.2.2. Proteins

Gelatin (GL) is a hydrophilic biopolymer, which has been widely used in the biomedical field [173]. Nanofibrous material based on GL was proposed as a carrier of active ingredients gained from natural sources. Specifically, electrospinning of GL in the presence of a small amount of the *Phaeodactylum tricornutuma* extracts provided a device with remarkable antimicrobial properties against *E. coli* and multidrug-resistant *S. aureus* [133,174]. Similarly, *Centella asiatca*, a traditional herbal medicine able to facilitate the wound-repair process, was involved in the fabrication of GL-based nanofibrous mats [175]. This device exhibited remarkable antimicrobial properties against *S. aureus*, *E. coli,* and *P. aeruginosa* and dermal wound-healing activity in the rat model. More recently, GL fibers loaded with *Curcuma comosa* Roxb. extract displayed antibacterial activities against *S. aureus* (Inhibition zone of 7.77 mm) and *S. epidermidis* (Inhibition zone of 7.73 mm) [135], while the loading of *Chromolaena odorata* crude extract [136] allowed the fabrication of a system with excellent antimicrobial activity against *S. aureus* (100% of inhibition). However, nanofibers only based on natural polymers, such as GL, are often not useful for biomedical applications due to their low mechanical strength and high rate of degradation [103]. Baghersad et al., (2018) fabricated hybrid scaffolds, based on GL, PCL, and aloe vera, as an active extract, displaying good antibacterial activity against *E. coli* (inhibition 85.63%) and *S. aureus* (inhibition > 99%) [137].

Silk fibroin (SF) nanofibers were largely employed in the biomedical field due to their valuable properties, such as biocompatibility, electrospinnability, low inflammatory response, and therapeutic features [176]. SF/PEO nanofibers, incorporating Manuka honey [177], have been successfully fabricated by electrospinning and evaluated as a potential antimicrobial tissue engineering scaffold [138]. Manuka honey/SF nanofibers exhibited antibacterial properties against methicillin-resistant *S. aureus*, *P. aeruginosa*, *E. coli,* and *S. aureus*. Similarly, SF/PVP nanofibers loaded with baicalein, a Chinese herbal extract, were prepared by electrospinning technique and proposed as wound healing devices [139]. Experimental release displayed that almost 65% of baicalein was delivered within 24 h, reaching the lag phase after 48 h. In addition, in vitro antibacterial test against *S. aureus* displayed complete inhibition of the pathogen, while in vivo experiments in mice treated with SFP/PVP/baicalein exhibited a significant acceleration of the wound closure process. SF was employed with hyaluronic acid to fabricate nanofibers by coaxial electrospinning [140], an innovative synthetic methodology able to produce nanofibers with sheath/core morphology, utilizing two needles that, coaxially placed, allowed to feed core and shell solutions throughout two different channels. Specifically, the device was structured by a shell of SF, while hyaluronic acid and olive leaf extract, a source of bioactives, formed the core. The analysis of the release profiles showed an almost complete delivery (>90%) in nine days, reaching the lag time after two weeks. Olive leaf extract-loaded nanofibers also revealed remarkable antibacterial features against *S. aureus* and *E. coli* bacteria. The same synthetic methodology was proposed to fabricate core-shell nanofibers from zein (core) and tragacanth gum (shell) [141] for the encapsulation of saffron extract [175,178]. Release values in the range 16.1–43.9% after 2 h were recorded in saliva, water, and in media, simulating gastric and intestinal fluids. Zein nanofiber mats loaded with ethanol propolis extracts were also effectively fabricated by the classic electrospinning method, and their antimicrobial activities were investigated against various microorganisms, highlighting that the nanofibers were able to mainly inhibit the growth of the Gram-positive species [142].

Finally, electrospun nanocomposite fibers were fabricated, employing soy protein isolate, PEO, and raspberry extract, a natural mixture containing high levels of anthocyanin, displaying a significant growth inhibition of *S. epidermidis* [173].

## 5. Nanoparticles as Carriers of Antimicrobial Natural Products

Several examples are present in the literature, describing the involvement of nanoparticles in chemical reactions to form new effective carriers to deliver antimicrobial agents from natural sources in order to eliminate pathogens without introducing chemical undesirable preservatives (Table 5) [179,180].

**Figure 3:**
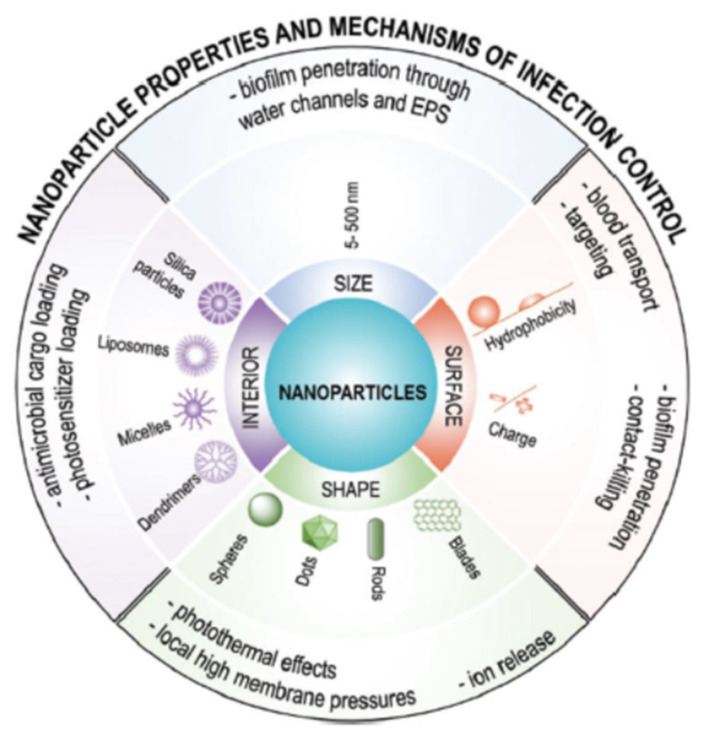
Size, shape, surface, and interior properties of nanoparticles important for their use in infection control. Reproduced with permission from [5], RSC, 2019.

The different methodology can be involved in the fabrication of nanoparticles with suitable properties, including soft lithography, mechanical stretching, microfluidics, or self-assembly, using suitable starting materials, such as small molecules or polymeric structures. When loading bioactive molecules into nanoparticle carriers with the aim to control pathogens growth and/or to prevent infection, different physicochemical and mechanical parameters should be considered, including size, shape, surface, and interior properties (Figure 3).

Nanoparticles size plays a crucial role in their penetration into biofilm-bacteria, ensuring to overcome the inconvenience usually related to the multidrug-resistant strains. Ideal nanoparticle sizes to fight bacterial infections range between 5 and 500 nm. Generally, to improve the nanocarriers’ efficiency, their size should not exceed the dimensions of water-filled channels into biofilms. In addition, nanoparticles sizes above 500 nm can be easily recognized by the immune system and eliminated from the bloodstream [5]. Surface properties should be also considered during nanoparticle design to ensure the inhibition of bacteria proliferation. Specifically, stealth transport through the bloodstream can be accomplished by decorating nanoparticles with suitable uncharged (polyethylene glycol) or zwitterionic hydrophilic polymers. Contact-killing capacity is strongly influenced by the nanoparticle shape that affects local adhesion forces and subsequently the extent of damage to the bacterial membrane [5]. In this regard, nano-blades or nano-knives by puncturing bacteria membranes determine the dispersion of intracellular components and cell death. Finally, the hydrophobic/hydrophilic balance into the interior of the nanoparticles should be designed to guarantee high loading capacity towards antimicrobial substances, avoiding the unnoticed loss of the cargo on their way to the infection site.

Cinnamon bark extract was embedded in PLGA/PVA nanoparticles synthesized by an emulsion evaporation method using different ratios of lactide/glycolide (65:35 and 50:50); TEM images revealed that nanoparticles were all spherical with a darker perimeter on the edge of the spheres attributed to the PVA. These products were able to gradually release active molecules and effectively inhibit *S. enterica* serovar Typhimurium and *L. monocytogenes* after 24 and 72 h at concentrations ranging from 224.42–549.23 μg/mL (Table 4) [181]. Acerola, guava, and passion fruit by-product extracts were also embedded in PLGA/PVA nanoparticles with a spherical shape and smooth surface. The antimicrobial performances against *L. monocytogenes* Scott A and *E. coli* K12 were concentration and time-dependent, with MIC values ranging from 200–1000 μg/mL, higher than the corresponding isolated extracts (from 500–3000 μg/mL) [182]. Passion fruit by-products (seed and cake) extracts were encapsulated into nanospheres synthesized by emulsion solvent evaporation method, involving different PLGA (lactide to glycolide ratios equal to 50:50 and 65:35) as organic phase and PVA aqueous solution (0.5 % *w/w*). The antimicrobial properties of the systems were evaluated against *E. coli* and *L. innocua*, and PLGA 65:35 particles showed a MIC value of 188 µg/mL against *L. innocua*, while the best PLGA against *E. coli* was PLGA 50:50 particle with a MIC of 226 µg/mL. In all the cases, the extracts were derived from cake extracts [183].

Similarly, PLGA/PVA nanoparticles (size range from 145–162 nm) were encapsulated with guabiroba extract (GE), a rich source of polyphenols and carotenoids. Release experiments, performed at 37°C and neutral pH, displayed an initial burst effect, more pronounced decreasing the lactide/glycolide ration in the PLGA chain, followed by a slower release rate of carotenoids. Nanoparticles based on PLGA 65:35 released 77% of the enclosed molecules after 6 h, while PLGA 50:50 displayed a marked burst effect (92% after 1 h). This behavior was a consequence of the highest lactide content able to delay the diffusion of the lipophilic carotenoids through the polymeric chains. Nanoparticles showed the growth inhibition of *L. innocua* within the concentration range tested (<1200 mg/mL), not displayed by the free extract [184]. The same authors improved the synthetic methodologies to achieve polymeric nanoparticles, proposing a modified emulsion-evaporation encapsulation method, allowing the synthesis of delivery devices able to preserve the extract’s phenolic content for a prolonged time during release. In this study, GE showed improved antioxidant efficacy and an interesting MIC value of 2.251 µg/mL for PLGA 65:35 nanoparticles against *L. innocua* [185].

In the search for new antibacterial materials, silica mesoporous nanoparticles (SMN) have also attracted burgeoning attention due to high surface area, good biodegradability, high biocompatibility, tunable pore/particle size, and easy surface functionalization [186]. In addition, the presence of a significant porous structure allows designing high-performance devices to be applied as a carrier in the pharmaceutical and biomedical fields. Sol-gel chemistry is the synthetic strategy usually proposed to prepare SMN, mainly involving two reaction steps: (i) synthesis of silica precursor around a template by reactions of hydrolysis and condensation; (ii) removing of the template by solvent extraction and calcination [187]. The synthetic strategy deeply determined physicochemical and the morphological properties of the SMNs, such as size, porosity, and surface properties, as well as their cytotoxicity and biocompatibility, which represent important peculiarity to their employment in biological fluids. In this regard, SMNs are hydrolyzed to the nontoxic silicic acid, easily and safely excreted, and/or absorbed by the human body [188].

By using tetra-alkylammonium and pluronic surfactants with different molecular weights, it was possible to easily tune SMN pore sizes to better control loading capacity and cargo release rate. In addition, a relevant concentration of silanol groups on the SMNs surface permitted the preparation of hybrid inorganic/organic nanodevices, mainly through condensation reactions, grafting methodology, or direct incorporation of organic moieties into the silica wall [189].

MCM-41 represents a member of SMN with specific morphological and structural properties: uniform hexagonal array and channels with pores ranging from 2–50 nm [190]. This structure was proposed as a carrier of red propolis [191], showing remarkable antibacterial, antioxidant, and anticancer properties [192]. MCM-41 nanocarrier was fabricated by the co-condensation method using n-cetyl-n,n,n-trimethyl ammonium bromide as a template. In this view, different amounts of red propolis were embedded in silica mesoporous nanoparticles and tested against *S. aureus* at concentrations of 1050, 750, 500, 375, 225, and 150 μg/mL, finding an inhibition zone of approximately 19, 20, 21, 17, 16, and 17 mm in diameter, respectively.

MCM-41 was also synthesized by the sol-gel method assisted by hydrothermal treatment and employing tetraethyl orthosilicate and n-cetyl-n,n,n-trimethyl ammonium bromide as silica source and template, respectively. Polyphenolic extracts of *Salvia officinalis* L. and *Thymus serpyllum* L. were encapsulated in these matrices and tested against *S. enterica*, *S. flexneri serotype 2b*, *E. faecalis*, *E. coli*, *P. aeruginosa*, *S. aureus*, *S. pneumoniae*, *S. pyogenes*, *B. fragilis*, *C. albicans,* and *C.n parapsilosis*, although showing milder improved antibacterial properties with respect to the parent extracts [193].

## 6. Conclusions and Future Perspectives

Nanotechnologies have been proposed as a valuable tool to overcome the problems frequently related to the employment of natural products with pharmacological activities. Indeed, most of the clinical trials involving natural products fail due to their poor water solubility, unsuitable molecular weight, and low lipophilicity, which produce unstable structures undergoing high metabolic rate and fast clearance. In addition, if the bioactive molecules are accumulated in non-targeted tissues and organs, significant side effects can be detected.

Currently, different studies proposed polymeric nanocarriers as favorable devices to increase bioavailability and activities of natural products, representing a plausible approach for the treatment of a variety of diseases. In particular, the perspectives of a multidisciplinary approach, expecting the combination between nanotechnology-based delivery systems and antimicrobial features from natural extracts, are promising in the infection control in order to produce multi-component devices able to avoid the multidrug-resistance, usually associated with the infections. Nanodevices, such as nanofibers or nanoparticles, were effectively proposed as carriers of extracts from plants, herbals, and agro-food by-products with remarkable antimicrobial properties.

Electrospun nanofibers, usually fabricated by single-fluid electrospinning, represent a valuable approach to produce high performing nanodevices. This goal was reached by employing both natural and synthetic polymers. However, the best results were recorded, fabricating blended macromolecular structures able to conjugate the feature of synthetic constituents (cheapness, mechanical resistance, and electrospinnability) with the highest biocompatibility of the natural polymers, reducing the adverse effects, potentially affecting the different biological compartments. In addition, the loading of several bioactive molecules within nanofibers required the optimization of different key factors, such as burst release reduction and/or low delivery efficiency. Recently, coaxial electrospinning was proposed as an innovative tool to fabricate core-shell nanostructures able to optimize the distribution of bioactive molecules according to the required function. In this way, the burst effect was highly reduced, and the antimicrobial effect against both Gram-positive and Gram-negative pathogens was recorded.

Alternatively, polymeric nanoparticles represent a valuable device with tailored physicochemical features to become a therapeutic revolution against human pathogens. They have shown to be biocompatible, non-toxic, safe, biodegradable, and easily eliminated, providing many advantages to release natural molecules with antimicrobial activity.

However, the transfer of these polymeric systems from the laboratory to practical healthcare applications remains a significant obstacle. In general, rigorous protocols of validation of in vitro and in vivo procedures are required to simplify translation from the bench to the clinical trials. Nowadays, some formulations are in clinical or preclinical stages in order to verify long-term toxicity, as well as degradation and metabolism. In the future, the clinical potential of these complex nanostructures should be deeply investigated, employing effective devices in the pharmaceutical and biomedical fields as delivery systems and/or tissue engineering scaffolds, useful to ensure infection control, to speed up skin regeneration, enhancing, in this way, patient’s quality of life.

Similarly, the challenges for large scale fabrication necessitate novelty from engineers and chemists, and regulatory policies have to facilitate access to trials and patients. Thus, employing these nanocarriers in clinical practice remains challenging and will represent a major focus in the next decades.

## Figures and Tables

**Table 1:** Plant extracts endowed with antimicrobial activity.

Source	Microorganisms	Antibacterial Activity	Ref.
Male date palm flower	*Pseudomonas savastonoi, Escherichia coli, Salmonella enterica, Agrobacterium tumefaciens, Bacillus subtilis, Staphylococcus aureus, Micrococcus luteus, Listeria monocytogenes*	10.5–12.1 ^a^	[33]
*Ferula ferulioides*	*S. aureus*	0.00025 > 0.128 ^b^	[34]
*Derris scandens*	*S. aureus, Bacillus cereus, E. coli, Pseudomonas aeruginosa*	0.06–13 ^b^	[35]
*C. aurantium* flowers	*B. cereus*	1.562 ≤ 6.250 ^b^	[36]
*Psidium sp., Mangifera sp.* and *Mentha sp*	*Streptococcus sanguinis*, *Streptococcus mutans*	31.63 ± 5.11 ^c^	[37]
*Pulicaria undulata* L	*S. aureus, E. coli, Klebsiella pneumoniae, P. aeruginosa*	17–18 ^d^	[38]
*Genista saharae*	*E. coli, Acinetobacter baumannii, Citrobacter freundii, Proteus mirabilis, Salmonella typhimurium, Enterobacter cloacae, S. aureus, B. cereus, B. subtilis, Enterococcus faecalis, L. monocytogenes*	0.01 > 1000 ^c^	[39]
*Cerbera manghas, Commelina diffusa, Kleinhovia hospita, Mikania micrantha, Omalanthus nutans, Peperomia pellucida, Phymatosorus scolopendria, Piper graeffei, Psychotria insularum, Schizostachyum glaucifolium*	*S. aureus, E. coli, P. aeruginosa*	0.004–0.512 ^b^	[40]
Fungi	−	−	[41]
*Psidium guajava* L. cv. Pearl	*E. coli, S. aureus, P. aeruginosa*	0.3 – 10.0 ^b^	[42]
*Polyscias scutellaria* Fosberg	*A.* sp.	225–400 ^d^	[43]
*Aspidosperma quebracho-blanco, Schinus fasciculatus, S. gracilipes, Amphilophium cynanchoides, Tecoma stans*	*Pseudomonas corrugate, Pseudomonas syringae pv. tomato, Erwinia carotovora var. carotovora, A. tumefaciens, Xanthomonas campestres pv. vesicatoria*	2.2 > 4.0 ^b^ 2.0–4.8 ^a^	[44]
Cardamom *(Elettaria cardamomum)*	*Aggregatibacter actinomycetemcomitans*, *Fusobacterium nucleatum*, *Porphyromonas gingivalis*, *Prevotella intermedia*	0.06–1.00 ^b^	[45]
*Origanum vulgare, Salvia officinalis, Thymus vulgaris*	*E. coli, Klebsiella oxytoca, K. pneumoniae*	2–370 ^b^	[46]
*Myristica fragrans*	*S. aureus,* methicillin-resistant *S. aureus, Streptococcus pyogenes, P. aeruginosa, Candida albicans*	0–12 ^b^ 0–45 ^a^	[47]
*Euphorbia tirucalli* L.	*S. aureus, Staphylococcus epidermidis, E. faecalis, E. coli, P. aeruginosa*	12.8–16.0 ^b^	[48]
*Tradescantia zebrina*	*B. cereus, B. subtilis, M. luteus, S. aureus, S. epidermidis*	5 > 10 ^b^	[49]
*Adiantum caudatum*	*B. subtilis, E. coli, P. aeruginosa*	8-22 ^a^	[50]
*Agastache rugosa* Korean Mint	*Aeromonas salmonicida, Cronobacter sakazakii, E. coli, Staphylococcus haemolyticus, Aeromonas hydrophila*	9.3–28.3 ^a^	[51]
Algae and diatoms	*E. faecalis, S. aureus, S. epidermidis, Streptococcus agalactiae, Streptococcus pneumoniae, S. pyogenes, Acinetobacter lwoffii, E. coli, K. oxytoca, K. pneumoniae, P. mirabilis, P. aeruginosa, Serratia marcescens*	6.0–12.0 ^a^ 0.062 > 1000 ^b^	[52]
*Fagus sylvatica* L	*S. aureus, P. aeruginosa, S. typhimurium, E. coli, Candida*	1-3 (MIC) ^b^; 3-6 (MBC) ^b^	[53]

^a^ Inhibition zone; ^b^ minimal inhibitory concentration (MIC), Minimum bactericidal concentration (MBC) (mg/mL); ^c^ cell-surface hydrophobicity of bacteria (%); ^d^ inhibition zone dimension (IZD) (mm).

**Table 2:** Agro-food wastes with antimicrobial properties.

Source	Microorganisms	Antimicrobial Activity	Ref.
Apple seeds	*Escherichia coli, Salmonella sp., Bacillus subtilis, Staphylococcus aureus, Candida sp., Saccharomyces cerevisiae, Aspergillus flavus, Penicillium citrinum, Mucor sp., Rhizopus sp.*	0.3–0.6 ^a^	[54]
Tomato peels	*S. aureus, B. subtilis, Listeria monocytogenes, E. coli, Pseudomonas aeruginosa, Salmonella typhimurium*	2.5–10.0 ^a^	[55]
Leaves of fennel and carrot	*Salmonella enteritidis, S. aureus, Candida albicans*	6.5–50.0 ^a^	[56]
Betel leaf stalk	*B. subtilis, E.coli, P. aeruginosa, S. aureus*	0.025–0.250 ^a^	[57]
Olive mill waste	*S. aureus*, *E. coli, Staphylococcus faecalis*	11.1–28.8 ^b^	[58]
Seed and peel of Citrus sinensis	*S. aureus, C. albicans*	2.5–40.0 ^a^ 2.0–14.0 ^b^	[59]
Orange peels of Citrus senensis	*S. aureus, L. monocytogenes, P. aeruginosa*	15–92 ^a^	[60]
Grape seeds	*E. coli, S. aureus*	9–21 ^b^	[61]
Grape pomace	*S. aureus, E. coli, C. albicans*	0.195–100 ^a^	[62]
Lavender (*Lavandula angustifolia*) and melissa (*Melissa Officinalis*) waste	*E. coli, Proteus vulgaris, P. aeruginosa, S. aureus, Enterococcus faecalis, L. monocytogenes, Candida utilis, B. subtilis, Aspergillus niger, Penicillium chrysogenum, S. cerevisiae*	8.00–12.00 ^a^	[63]
Mango seed kernel	*Xanthomonas axonopodis pv. manihotis*	3.08–7.10 ^b^	[64,65]
	*E. coli, C. albicans*	1.10–2.23 ^a^	
*Vaccinium meridionale* Swartz pomace	*S. aureus, E. coli*	126–520 ^c^	[66]
Walnut green husk	*Bacillus cereus, B. subtilis, S. aureus, Staphylococcus epidermis, E. coli, P. aeruginosa*	20–100 ^a^	[67]
Carya illinoinensis	*L. monocytogenes, S. aureus, Vibrio parahaemolyticus, B. cereus*	0.075–1.870 ^a^	[68]
Garlic (*Allium sativum L.*) husk	*P. aeruginosa, Klebsiella pneumoniae*	1–10 ^a^	[69]
Mangosteen bark, leaf, and fruit pericarp	*L. monocytogenes, S. aureus*	0.03 > 10 ^a^	[70]
Newhall navel orange peel	*E. coli, S. aureus, B. subtilis*	0.16–30.36 ^a^	[71]
Peel of *Punica granatum* Var. Bhagwa	*S. aureus, E. coli, Streptococcus mutans mutans, C. albicans*	17–32 ^a^	[72]
Brewers’ spent grain	*S. aureus L. monocytogenes, S. typhimurium, E. coli, P. aeruginosa, C. albicans*	0.00097–0.125 ^a^	[73]
*Agave sisalana* Perrine juice (waste)	*E. faecalis, C. albicans, P. aeruginosa, Bacillus atrophaeus, Shigella dysenteriae*	24–31 ^b^	[74]
Orange, yellow lemon, and banana peel	*P. aeruginosa, K. pneumoniae, Serratia marcescens, E. coli, P. vulgaris, Salmonella typhi, S. aureus, E. faecalis, Aeromonas hydrophila, Streptococcus pyogenes, L. monocytogenes, Lactobacillus casei*	9–35 ^b^	[75]
Guava bagasse (*Psidium guajava*), Cabernet Sauvignon, Pinot Noir (*Vitis vinifera*), Isabella grape marcs (*Vitis labrusca*), Petit Verdot grape seeds and red grapes fermentation lees (*Vitis vinifera*), tomato bagasse (*Solanum lycopersicum*), kale (*Brassica oleracea*), beet (*Beta vulgaris*), broccoli (*Brassica oleracea*), turnip stems (*Brassica rapa*), carrot (*Daucus carota*), radish leaves (*Raphanus sativus*), pumpkin (*Cucurbita* sp.), passion fruit hulls (*Passiflora edulis*), artichoke leaves (*Cynara cardunculus*), and peanut peels (*Arachis hypogaea*)	*S. aureus, L. monocytogenes, S. Enteritidis, E. coli*	10.0–20.0 ^d^ 0.78–25.00 ^a^	[76]
Mango (*Mangifera indica* L.),	*B. subtilis, S. aureus, P. aeruginosa, E. coli*	13–18 ^b^	[77]
Camu-camu (*Myrciaria dubia* (Kunth) McVaugh)	*E. coli, K. pneumoniae, Morganella morganii, Proteus mirabilis, P. aeruginosa, E. faecalis, L. monocytogenes*	0.625 > 20	[78]
Olive mill wastewater	*Campylobacter* strains	0.25–2.00 ^a^	[79]
Coffee pulp and husk	*Salmonella cholerasus, S. aureus, P. aeruginosa, L. monocytogenes, E. coli*	0.000612–0.001225 ^a^	[80]

MIC = minimum inhibitory concentration; MBC = minimum bactericidal concentration; IZ = inhibition zone; GAE = gallic acid equivalent. ^a^ MIC/MBC (mg/mL); ^b^ disc diffusion method (mm); ^c^ μg GAE/mL; ^d^ IZ (inhibition zone mm).

**Table 3:** Synthetic polymers employed in the fabrication of nanofibers for the delivery of antimicrobial natural extracts.

Nanofiber	Incorporated Species	Antimicrobial Activity Against	Applications	Ref.
	Moringa leaf extracts	*Staphylococcus aureus, Escherichia coli*	Wound dressing	[92]
Polyacrylonitrile	*Syzygium aromaticum* oil	*S. aureus, Bacillus subtilis, E. coli, Klebsiella pneumoniae*	Wound dressing and tissue engineering scaffolds	[93]
Polyacrylonitrile	Lavender essential oil	*S. aureus, K. pneumoniae*	Antimicrobial activity	[94]
Poly (lactic acid)	*Leptospermum scoparium* and *Melaleuca alternifolia* essential oil	*Staphylococcus epidermidis*	Tissue engineering scaffolds	[95]
Poly (d,l-lactide-*co*-glycolide)	Methanolic extract of *Grewia mollis*	*S. aureus, E. coli*	Wound dressing	[96]
Poly (d,l-lactide-*co*-glycolide)	Human epidermal growth factor and *Aloe vera* extract	*S. aureus, S. epidermidis*	Wound dressing	[97]
Polycaprolactone	*Biophytum sensitivum* extract	*S. aureus*, *E. coli*	Wound dressing	[98]
Polycaprolactone	*Gymnema sylvestre*	*Pseudomonas aeruginosa*, *E. coli*	Wound dressing	[99]
Polycaprolactone	*Clerodendrum phlomidis*	*S. aureus, P. aeruginosa, Salmonella typhi*, *E. coli*	Wound dressing	[100]
Polycaprolactone, gelatin	*Althea officinalis*	*−*	Tissue engineering scaffolds	[101]
Polycaprolactone, gelatin	Black pepper oleoresin	*S. aureus*	Antimicrobial activity	[102]
Polycaprolactone, polyvinyl pyrrolidone	*Tecomella undulate* extract	*P. aeruginosa, S. aureus, E. coli*	Wound dressings	[103]
Polycaprolactone, silver nanoparticles	*Nepelium lappaceum* extract	*E. coli, S. aureus, P. aeruginosa*	Wound dressings	[104]
Polyurethane	Emu oil	*B. subtilis, E. coli*	Wound dressings and tissue engineering scaffolds	[105]
Polyurethane	Propolis	*E. coli*	Wound dressings and tissue engineering scaffolds	[84]
Polyurethane	*Agrimonia eupatoria*, *Satureja hortensis*, *Hypericum perforatum* herbal extract	*S. aureus*, *P. aeruginosa*	Wound dressings	[106]
Polyurethane	*Szygium aromaticum* extract	*S. aureus, E. coli*	Wound dressings	[107]
Polyurethane/polyethylene terephthalate	*Juniperus chinensis* extract	*S. aureus*, *K. pneumoniae*	Antimicrobial clothing materials, bedding materials	[108]
Polyurethane/carboxymethyl cellulose	*Malva sylvestris* extract	*S. aureus, E. coli*	Wound dressings	[109]
Poly (vinyl alcohol)	*Lawsonia inermis* leaves extract	*S. aureus, E. coli*	Wound dressings	[110]
Poly (vinyl alcohol)	*Tridax procumbens* leaves extract	*S. aureus, E. coli*	Antimicrobial activity	[111]
Poly (vinyl alcohol)	*Coptis chinensis* extract	*S. aureus*, *S. epidermidis*	Medical and cosmetics fields	[112]
Poly (vinyl alcohol)	*Rhodomyrtus tomentosa* extract	*E. coli, P. aeruginosa, B. subtilis*, *Enterococcu faecalis*	Antimicrobial activity	[113]
Poly (vinyl alcohol)	*Coptidis rhizoma* extract	*S. aureus*, *S. epidermidis*	Wound dressings	[114]
Poly(vinyl alcohol)/nanocellulose from pineapple	*Stryphodedron barbatimao* extract	*−*	Antimicrobial activity	[115]
Poly(vinyl alcohol)/guar gum	*Acalypha indica* extract	*E. coli*, *B. subtilis*, *S. aureus*, *Pseudomonas fluorescens*	Wound dressings	[116]
Poly(vinyl alcohol)/sodium alginate	Cinnamon, clove, and lavender oils	*S. aureus*	Wound dressings	[117]
Polyvinylpyrrolidone	*Sophora flavescens* extract	*S. epidermidis*	Antimicrobial air filtration	[118]
Polyvinylpyrrolidone	Cinnamon essential oil	*S. aureus, E. coli, P. aeruginosa, Candida albicans*	Antimicrobial activity	[119]

**Table 4:** Natural polymers employed in the fabrication of nanofibers for the delivery of antimicrobial natural extracts.

Nanofiber	Incorporated Species	Antimicrobial Activity Against	Applications	Ref.
Cellulose acetate	Lemongrass, cinnamon, and peppermint essential oils	*Escherichia coli*, *Candida albicans*	Tissue engineering scaffolds	[120]
Cellulose acetate	Rosemary and oregano essential oils	*Staphylococcus aureus, E. coli, C. albicans*	Wound dressings	[121]
Carboxymethyl cellulose/sodium alginate	Olive leaf extract	*S. aureus, E. coli, Enterococcus faecalis, Pseudomonas aeruginosa*	Wound dressings	[122]
Cellulose	*S. persica* extract	*S. aureus, E. coli*	Antimicrobial activity	[123]
Cellulose	*Oryza sativa* and *Tinospora cordifolia* extracts	*E. coli*, *P. aeruginosa*, *Bacillus subtilis*	Wound dressing and tissue engineering scaffolds	[124]
Dandelion polysaccharide	*Litsea cubeba* essential oil	*S. aureus*	Antimicrobial activity	[125]
Chitosan-ethylenediaminetetra acetic acid/poly(vinyl alcohol)	*Garcinia mangostana* fruit hull extract	*S. aureus, E. coli*	Wound dressings	[126]
Chitosan/poly(vinyl alcohol)	*Bidens pilosa* extract	*S. aureus, E. coli*	Antimicrobial activity	[127]
Chitosan/poly(vinyl alcohol)/honey	−	*S. aureus, E. coli*	Wound dressings	[128,129]
Chitosan/poly(vinyl alcohol)/honey	*Cleome droserifolia* and *Allium sativum* extracts	Multidrug-resistant *P. aeruginosa*, *E. coli*, *S. aureus,* and methicillin-resistant *S. aureus*	Wound dressings	[130]
Chitosan/poly(ethylene oxide)	Cinnamaldehyde essential oil	*P. aeruginosa*	Tissue engineering scaffolds	[131]
Chitosan/poly(ethylene oxide)	Green tea extract	*S. aureus, E. coli*	Wound dressings	[132]
Gelatin	*Phaeodactylum tricornutuma* extract	Multidrug-resistant *P. aeruginosa*, *E. coli*	Wound dressings	[133]
Gelatin	*Centella asiatca*	*S. aureus*, *E. coli,* and *P. aeruginosa*	Wound dressings	[134]
Gelatin	*Curcuma comosa* Roxb. extract	*S. aureus*, *Staphylococcus epidermidis*	Antimicrobial activity	[135]
Gelatin	*Chromolaena odorata* crude extract	*S. aureus*	Antimicrobial activity	[136]
Gelatin/polycaprolactone	*Aloe vera* extract	*S. aureus, E. coli*	Tissue engineering scaffolds	[137]
Silk fibroin/poly(ethylene oxide)	Manuka honey	Methicillin-resistant *S. aureus*, *P. aeruginosa*, *E. coli*, *S. aureus*	Wound dressings	[138]
Silk fibroin/polyvinylpyrrolidone	Baicalein	*S. aureus*	Wound dressings	[139]
Silk fibroin/hyaluronic acid	Olive leaf extract	*S. aureus, E. coli*	Antimicrobial activity	[140]
Zein/tragacanth gum	Saffron extract	*−*	Polymeric carrier	[141]
Zein	Propolis extract	*S. aureus, S. epidermidis, E. coli, Salmonella enterica, P. aeruginosa, C. albicans*	Wound dressings	[142]
Soy protein isolate	Raspberry extract	*S. epidermidis*	Antimicrobial activity	[143]

**Table 5:** Synthetic polymers employed in the fabrication of nanoparticles for the delivery of antimicrobial natural extracts.

Nanoparticle	Incorporated Species	Antimicrobial Activity Against	Applications	Ref.
Poly(d,l-lactide-*co*-glycolide)/poly(vinyl alcohol)	Cinnamon bark extract	*Salmonella enterica* serovar Typhimurium, *Listeria monocytogenes*	Antimicrobial activity	[181]
Poly(d,l-lactide-*co*-glycolide)/poly(vinyl alcohol)	Acerola, guava, and passion fruit waste extracts	*L. monocytogenes* Scott A, *Escherichia coli* K12	Improvement of physical texture	[182]
Poly(d,l-lactide-*co*-glycolide)/poly(vinyl alcohol)	Passion fruit by-products	*E. coli*, *Listeria innocua*	Chemical disinfectants	[183]
Poly(d,l-lactide-*co*-glycolide)/poly(vinyl alcohol)	Guabiroba extract	*L. innocua*	Delivery systems	[184,185]
Silica mesoporous nanoparticles	Red propolis	*Staphylococcus aureus*	Antimicrobial activity	[191]
Silica mesoporous nanoparticles	*Salvia officinalis L.* and *Thymus serpyllum L.*	*S. enterica*, *Shigella flexneri serotype 2b*, *Enterococcus faecalis*, *E. coli, Pseudomonas aeruginosa*, *S. aureus*, *Streptococcus pneumoniae*, *Streptococcus pyogenes*, *Bacteroides fragilis*, *Candida albicans*, *Candida parapsilosis*	Antimicrobial activity	[193]
